# National road traffic accident information system in Iran and selected countries

**DOI:** 10.5249/jivr.v17i1.1935

**Published:** 2025-01

**Authors:** Mahrokh Anvari, Hassan Emami, Azamossadat Hosseini, Ali Delpisheh, Farkhondeh Asadi

**Affiliations:** ^*a*^ Department of Health Information Technology and Management, School of Allied Medical Sciences, Shahid Beheshti University of Medical Sciences, Tehran, Iran.; ^*b*^ School of Health & Safety, Safety Promotion and Injury Prevention Research Center, Shahid Beheshti University of Medical Sciences, Tehran, Iran.

**Keywords:** Road safety, Crash data, Information system, Data exchange, Registration, Hospital data, Crash record, Organization

## Abstract

**Background:**

The National Road Traffic Accident Information System is crucial in enhancing road and traffic safety by providing managers and policymakers with systematic access to and analysis of crash data. Accordingly, the present study aims to review the data collection and exchange processes within these systems and to identify the roles and significance of the participating organizations.

**Methods:**

The current study was conducted in accordance with the Preferred Reporting Items for Systematic Reviews and Meta-Analyses (PRISMA) guidelines. Comprehensive literature searches were carried out using Scientific Information Database (SID), Google Scholar, Science Direct, and PubMed with the publication date range restricted to 1995–2024. Screening and selection of the studies retrieved were performed based on predefined inclusion and exclusion criteria. The quality assessment of the studies included in this review was conducted using the Mixed Methods Appraisal Tool (MMAT) and the Critical Appraisal Skills Programme (CASP).

**Results:**

Of the 22 studies included, 10 (45%) focused on data collection and recording within the National Road Traffic Accident Information System. Seven studies (31%) examined the role and significance of the organizations involved with these systems, while five studies (22%) focused on the data exchange process. According to the MMAT evaluation criteria, 13 studies (81%) demonstrated a low risk of bias, indicating a high level of quality. In contrast, two studies (12%) showed a moderate risk of bias, and one study (6%) had a high risk of bias, meeting less than 50% of the assessed criteria. Based on the CASP evaluation of the six review studies, four (66%) showed a low risk of bias, while two (33%) exhibited a moderate risk of bias.

**Conclusions:**

The findings of this review highlight the critical importance of prioritizing a leading organization and delineating a standardized minimum crash dataset. This approach has the potential to streamline the data recording process, foster inter-organizational communication and coordination, mitigate the occurrence of contradictory reports, and enhance the overall effectiveness of decision-making.

## Introduction

Road traffic accidents represent a significant public health concern and are among the leading causes of death and injury globally.^1^ Official statistics indicate that approximately 1.2 million people lose their lives annually due to traffic accidents, while nearly 50 million sustain injuries.^2^ In Iran, traffic accidents result in over 23,000 fatalities and injuries each year.^3^ Consequently, road transportation safety has become a critical focus in the country’s policies and strategic plans.^4^ Compared to many other nations, Iran experiences a disproportionately high rate of injuries and fatalities from road accidents, necessitating the implementation of targeted interventions.^5^ Effectively addressing this issue requires implementing an integrated information system to identify road safety challenges and the contributing factors.^6,7^ The development of a National Road Traffic Accident Information System offers several critical benefits, including streamlined data registration, reduced costs associated with data storage and analysis, enhanced coordination and communication among relevant organizations, prevention of contradictory reporting, problem identification, and support for informed decision-making processes.^8-10^ Furthermore, utilizing such a system allows stakeholders to achieve these advantages by leveraging existing data, eliminating the need for new data collection, which can often be time-consuming and costly.^11^

The National Road Traffic Accident Information System comprises a network of locations (nodes) and connections (links) that represent the relationships between these locations.^7,12^ The nodes within this system include individuals and organizations that play a vital role in enhancing performance and mitigating the negative impacts of road accidents.^13-15^ Additionally, the system incorporates databases where essential data must be accurately collected and registered. Effective data registration and exchange between these databases rely on well-defined data elements that represent the system and its key attributes.^16,17^ Through this system, spatial features, environmental factors, geographical characteristics, and the geometric design of roads can be analyzed—factors that significantly influence accident prevention.^18^

The exchange and registration of data among road traffic crash databases enable researchers to analyze crash characteristics, identify variables influencing injury severity, and examine variations based on crash types. This information is critical for reducing at-risk populations and addressing disparities.^11^ To explore these aspects, the current study examines the National Road Traffic Accident Information Systems in the United States, England, and Malaysia. For instance, the National Highway Traffic Safety Administration (NHTSA) in the United States provides safety data across three levels: local, state, and federal. At the state level, police accident reports and road transport injury data are collected and subsequently submitted to the Fatality Analysis Reporting System (FARS) database.^6^ The FARS is an online database that provides statistics on motor vehicle accidents.^19^ Similarly, in England, the police collaborate with the Department for Transport (DfT) to collect data on road accidents.^20^ The Collision Recording and Sharing (CRASH) system in England facilitates the collection, validation, transfer, and storage of road accident reports, tailored to meet the operational needs of the police. This system enhances the efficiency of police workflows, enabling the production of accurate and timely data.^20,21^ In Malaysia, the Road Safety Department (RSD) is recognized as the primary authority on road and traffic safety. The Centralized System for Road Accident, Safety, and Hazard Studies (CRASH) serves as the national-level crash data management system. Its primary objective is to integrate and centralize spatial data.^22^ The Swedish Traffic Accident Data Acquisition (STRADA) system oversees the entire data management process, from collection to making the information accessible at both local and national levels.^23^ This system is managed by the Swedish National Road Administration (SNRA).^24,25^ In South Korea, the Korea Road Traffic Authority (KoROAD) developed the Traffic Accident Analysis System (TAAS) to facilitate the integration and exchange of data between various road accident databases, including those maintained by the police and insurance companies.^26^ In Iran, the Road Safety Commission is a legal entity responsible for overseeing road safety and traffic management. Operating under the supervision of the Ministry of Interior, it aims to establish coordination among various organizations.^16,27,28^ Given the critical role of a national road accident information system in recording and exchanging accident data, this research has been conducted to provide accurate information and enhance the awareness of researchers and stakeholders, enabling them to take necessary actions to improve this system in Iran.

## Methods 

Original articles published electronically were selected using the Preferred Reporting Items for Systematic Reviews and Meta-Analyses (PRISMA) guidelines. This study focused on the United States, Sweden, and England due to their advanced and integrated accident data information systems.^29-34^ Malaysia and South Korea were included as representative Asian countries to examine their road traffic accident information systems.^34-36^ The review categorized the articles related to the National Road Traffic Accident Information System into three groups based on their focus: the importance of organizations, the registration process, and data exchange.

**Search strategy**

Relevant studies published between 1995 and 2024 were retrieved from the Science Direct, PubMed, Google Scholar, and SID databases. The search strategy was developed using common keywords identified in the literature on road traffic accident information systems (Table 1). The keywords included synonyms for terms such as "road safety", "crash data", "information system", "organization", "data exchange", "registration", "hospital data", and "crash record". A combination of logical operators "OR" and "AND" were employed to construct an effective search query. Additionally, manual searches were conducted using the Google search engine with relevant terms. Reference citations from selected articles were also reviewed to identify further studies.

**Table 1:** Search strategy

Section	Sub-section	Particularity
Keywords	Core keywords	registration; hospital data; crash record; data exchange; road safety; crash data; information system; organization
	Supplementary keywords	highway; car; report; crash; fatality; transportation; traffic; accident; motor-vehicle; injury; hospi-tal; emergency service; system; collection; information; database; linkage ; integrated; lead or-ganization; collision
Operators		“OR”, “AND”
Search formula		((“car accident” OR “automobile accident” OR “motor accident” OR “car crash” OR “travel acci-dent” OR “traffic accident” OR “traffic injury” OR “traffic trauma” OR “traffic damage “transport accident” OR “motor-vehicle ” OR “transport crash” OR “traffic crash” OR “traffic collision” OR “car collision” OR “transport fatal” OR “road fatal” “traffic fatality” OR “police” OR “police re-port”) AND (hospital OR “hospital-based” OR “hospital report” OR “department emergency” OR “emergency” OR “urgency”) AND ( data exchange” OR “exchanging” OR “ data exchanged” OR “data exchange” OR “record exchange” OR “ information exchange” ) AND ( “data base” OR “information system ” OR “system ” OR “accident information system ” crash information system ” OR “ crash system ” ) AND ( “linkage” OR “data linked ” OR “report linked ” OR “record linked ” linking ambulance ” OR “ linking hospital ” ) AND (“registration ” OR “data collection” OR “death registry” OR “recorded”) AND (“organization” OR “institution” OR “management ” OR “coordina-tion ” OR “organizing ” OR “top organization ”) AND (“road safety” OR “highway safety” OR “road safety system” OR “roadway safety” OR “road safety information system ” OR “street safety” OR “pathway safety information system”))
Time period		1995–2024

**Inclusion and exclusion criteria**

Selection for each article relied on the following predefined inclusion and exclusion criteria. 

**Inclusion criteria:**

• Studies with full-text availability.

• Studies providing a clear explanation of the registration process, data exchange mechanisms, and the role of accident-related organizations.

• Studies focusing on the National Road Traffic Accident Information System in the United States, England, or Malaysia.

**Exclusion criteria:**

• Studies with insufficient data on the National Road Traffic Accident Information System.

• Studies not addressing road traffic incidents or road safety.

**Screening and selection**

The initial screening of articles was conducted by two authors focusing on abstracts and titles of the papers. Subsequently, the selection of studies based on the full analysis was carried out by three authors independently, following the predefined inclusion and exclusion criteria. Any disagreements were resolved by a fourth author to ensure consistency and accuracy.

**Data extraction**

In the preliminary review, the abstracts of the articles were reviewed, and if deemed necessary, their full texts were also reviewed. Subsequently, the full texts of the relevant articles were reviewed and summarized. Key details regarding the methodology and results of the National Road Traffic Accident Information System were recorded using a standardized data extraction form. The extracted data elements included the authors' names, research title, country, publication year, research type, and key findings. The collected data were then analyzed using descriptive statistics.

**Quality assessment**

Systematic reviews following the PRISMA guidelines require a thorough appraisal of the included studies. The Mixed Methods Appraisal Tool (MMAT) serves as an effective instrument for assessing the procedural quality of articles of various designs covered in review articles.^37^ Accordingly, MMAT was employed to evaluate the risk of bias in the selected studies. Additionally, the Critical Appraisal Skills Programme (CASP) tool was used to measure the quality of review articles. The CASP tool adopts a similar approach, focusing on the rigor of systematic reviews.^38^ To ensure validity, two authors independently performed the quality assessment of the retrieved article. This process enabled the identification of studies with lower methodological standards. Since excluding such studies is not recommended, the analysis of research outcomes accounted for the potential impact of bias. 

## Results

**Systematic literature search**

**Figure 1:**
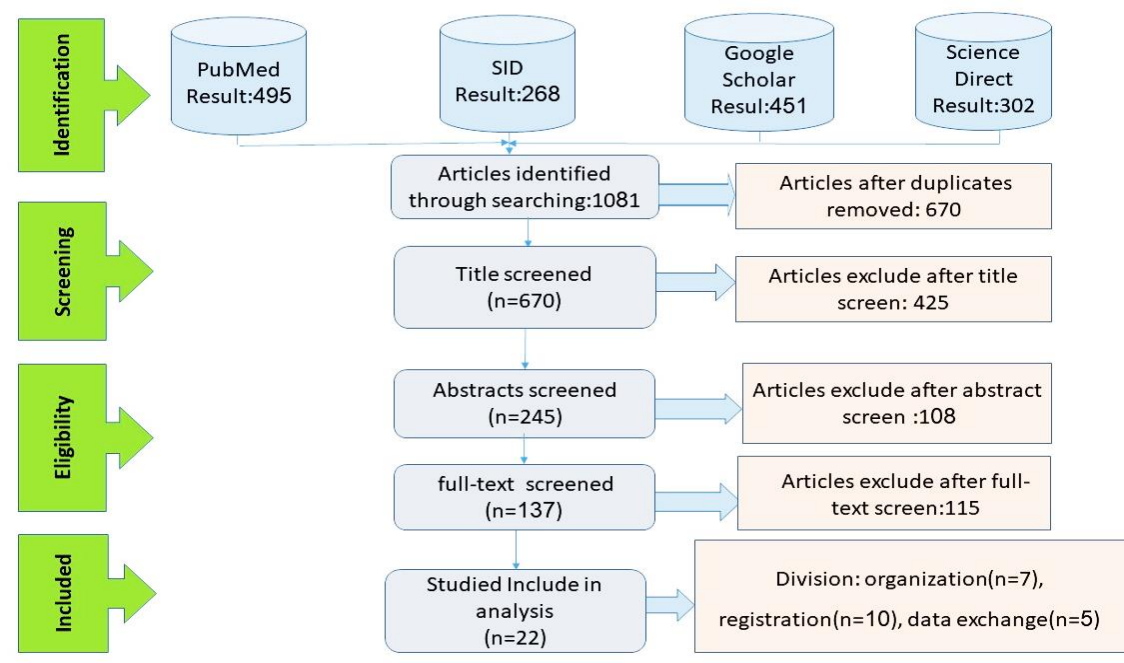
PRISMA diagram for the process of reviewing and removing articles

The preliminary search results from the four databases were stored in a centralized system. To identify and remove duplicates, the author names, titles, and publication years of each article were entered into the "Find" function, which facilitated the evaluation of titles, removal of duplicates, and screening of keywords and abstracts based on the inclusion and exclusion criteria outlined in Table 4. In total, 670 articles were retrieved from the databases. After an initial review of the abstracts, 245 articles were screened, followed by a full-text review of 137. Ultimately, 22 articles met the eligibility criteria and were included in the study. The PRISMA methodology guided the article selection process, as illustrated in Figure 1.

**Result of quality assessment**

A total of 16 studies (72%) were assessed using the MMAT checklist. The 16 studies included were categorized as follows: 4 qualitative studies (25%), three quantitative studies (18%), and nine mixed-method studies (56%). Based on the MMAT assessment, 13 articles (81%) met (75–100%) of the MMAT checklist, indicating high quality. Additionally, two articles (12.5%) met (50–75%) of the evaluated criteria, demonstrating moderate quality, while one article (6.25%) met less than (50%) of the evaluated criteria, reflecting low quality (Table 2). Furthermore, six review articles (27%) were evaluated according to CASP criteria. Among these, four articles (66%) were deemed high quality and two articles (33%) were categorized as moderate quality (Table 3).

**Table 2:** Quality assessment of the articles based on quality, quantity, and mixed methods, using the MMAT checklist

MMAT						
**Qualitative studies**						
**Reference**	**Is the qualitative approach suitable?**	**Are qualitative data collection methods sufficient?**	**Are the findings data-driven?**	**Is data adequately supporting results?**	**Is there coherence in qualitative data processes?**	**MMAT Rating**
Eusofe (2017)^13^	Yes	No	Yes	No	No	Low
Sadeghi (2020)^39^	Yes	Yes	Yes	Yes	Yes	High
Maleki (2018)^40^	Yes	Yes	Yes	Yes	No	Moderate
Ehsani (2023)^41^	Yes	Yes	Yes	Yes	Yes	High
**Quantitative studies**						
**Reference**	**Is the sampling strategy suitable?**	**Is the sample population representative?**	**Are the measurements appropriate?**	**Is nonresponse bias low?**	**Is the statistical analysis fitting?**	**MMAT Rating**
Kamaluddin (2019)^42^	Yes	Yes	Yes	No	Yes	High
Bakhtiari (2013)^43^	Yes	Yes	Yes	Yes	Yes	High
Stigson (2021)^35^	Yes	Yes	Yes	Yes	Yes	High
**Mixed method studies**						
**Reference**	**Is a mixed methods design justified?**	**Are the study components well integrated?**	**Are the integration outputs correctly interpreted?**	**Are divergences in results adequately addressed?**	**Do study components meet quality standards?**	**MMAT Rating**
Mohammadi (2016)^17^	Yes	Yes	Yes	No	Yes	High
Nie (2021)^44^	Yes	Yes	Yes	No	Yes	Moderate
Khorshidi (2015)^45^	Yes	Yes	Yes	Yes	Yes	High
Varhelyi (2016)^46^	Yes	Yes	Yes	No	Yes	High
Chung (2015)^47^	Yes	Yes	Yes	Yes	Yes	High
Sadeghi (2020)^12^	Yes	Yes	Yes	Yes	Yes	High
Hosseinzadeh (2022)^48^	Yes	No	Yes	Yes	Yes	High
Nazif-Munoz (2023)^49^	Yes	Yes	Yes	Yes	Yes	High
Han (2023)^50^	No	Yes	Yes	Yes	Yes	High

**Table 3:** Quality assessment of the review articles using the CASP checklist

CASP										
Review studies										
Reference	Did the review answer the question?	Did the authors seek relevant papers?	Were all key studies included?	Did the authors adequately assess quality?	Was combining the review results justified?	What are the review's overall results?	Are the results locally applicable?	Were all outcomes evaluated?	Are benefits greater than harms?	CASP Rating
Soltani (2024)^11^	Yes	Yes	Yes	Yes	Yes	Yes	Yes	Yes	Yes	High
Eskandari (2022)^51^	Yes	Yes	Yes	No	Yes	Yes	Yes	Yes	Yes	High
Rabbani (2022)^31^	Yes	Yes	Yes	No	No	Not clear	Yes	Yes	Yes	Moderate
Imprialou(2019)^30^	Yes	Yes	Yes	No	Yes	Yes	Yes	Yes	Yes	High
Abdul (2017)^19^	Yes	Yes	Yes	No	No	Not clear	Yes	Yes	Yes	Moderate
Wang(2013)^52^	Yes	Yes	Yes	No	Yes	Yes	Yes	Yes	Yes	High

**Characteristics of the included studies**

Among the 22 selected articles, seven articles (31.8%) were published in Iran,^11,12,17,39,40,43,45^ five articles (22.7%) in the United States,^19,41,44,48,49^ three articles (13%) in England,^30,51,52^ two articles (9%) in Sweden,^46,35^ two articles (9%) in South Korea, ^47,50,13,31,42^ and three articles (13%) in Malaysia.^13,31,42^ The studies were categorized based on their methodology: quantitative, qualitative, mixed-methods, and review studies. Among these, six articles (27%) were review studies.^11,19,30,31,51,52^ The majority, nine articles (56%), employed mixed methods,^12,17,44-50^ while four articles (18%) were qualitative.^13,39-41^ Another three articles (13%) were quantitative.^35,42,43^ A total of 18 articles (94%) were published between 2013 and 2024. In Iran, the largest group, four articles (21.3%), focused on crash data registration.^12,17,39,45^ Two articles (10.5%) in Iran addressed data exchange.^11,43^ The smallest number of articles, one study (5.2%) each, addressed the role of organizations in Iran, Malaysia, Sweden, and South Korea.^13,40,46,50^ In contrast, the United States had two studies (9%) that focused on the role of organizations in reducing traffic accidents and enhancing road safety^41,49^

**Figure 2:**
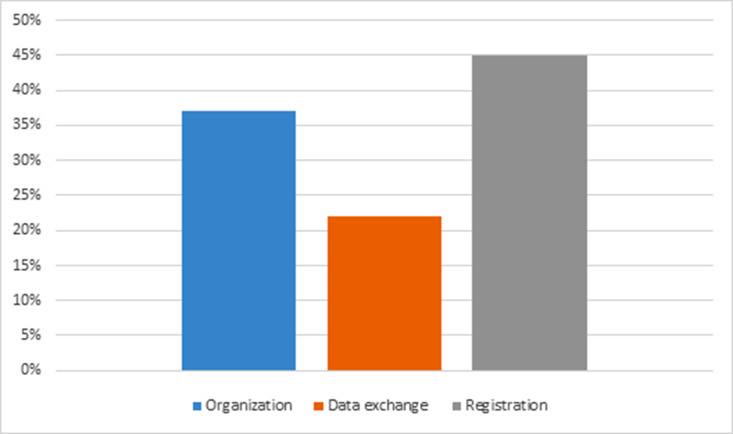
Percentage of studies conducted across three research groups

Table 4 shows that 10 articles (45%) focused on the importance of recording and collecting crash data. Accurate data recording is essential for generating consistent reports and statistics.^12,17,19,30,31,39,44,45^ Additionally, seven articles (37%) highlighted the importance of coordination between the involved organizations in reducing accidents.^6,13,40,41,25-54^ Another five articles (22%) emphasized data exchange as a critical component of the National Road Traffic Accident Information System^11,42,43,48,51^ (Figure 2).

**Table 4:** Details of the articles that were ultimately included in the review

Author's and year	Study Title	Country	Study Type	Research Group	Final findings
Ehsani JP, et al. (2023)^41^	The future of road safety: challenges and opportunities	USA	(Quantitative study)	Organization	The crash information system must evolve through collaboration between public and private organizations to ensure the safe and efficient movement of people and goods, without relying on private vehicle ownership, and in support of encouraging the use of public transportation.
Nazif-Munoz JI, et al. (2023)^49^	Assessing the impact of road safety agencies and health systems in traffic outcomes across 146 countries, 1994–2012	USA	(Mixed method study)	Organization	Collaboration and communication between road safety organizations and health institutions have a positive impact on reducing traffic-related injuries.
Wang ch, et al. (2013)^52^	The effect of traffic and road characteristics on road safety: A review and future research direction	UK	Review study	Organization	Various factors, including environmental characteristics, human behavior, and organizational relationships, contribute to the occurrence of accidents.
Eusofe Z, et al. (2017)^13^	Assessment of road safety management at institutional level in Malaysia	Malaysia	(Qualitative study)	Organization	The performance and effectiveness of road safety management can be enhanced based on government resources, the legal safety framework, public awareness, local needs, and the institutional capacity of the organizations involved.
Malek A, et al. (2018)^40^	Presenting the inter -organizational relations of traffic police in traffic safety. Military science and technology	Iran	(Qualitative study)	Organization	An investigation into the effectiveness of various governmental institutions in promoting road safety, with a focus on determining the proportion of influence each institution holds within the decision-making hierarchy.
Han S, et al. (2023)^50^	Improvement of road safety management systems of local governments in Korea after evaluating related indicators	South Korea	(Mixed method study)	Organization	Road safety management consists of institutional management, intervention and outcome. Road safety is managed not only in terms of results but also in terms of organizational accountability.
Varhelyi A, et al. (2016)^46^	Road Safety Management ‒ The Need for a Systematic Approach	Sweden	Review study	Organization	It is necessary to have a lead organization for road safety at the national level, and this could be a National Road Safety Commission consisting of relevant institutions (Justice, Ministry of Health, Education, Police, Industry, Transportation).
Abdulhafedh, A, (2017)^19^	Road Traffic Crash Data: An Overview on Sources, Problems, and Collection Methods	USA	Review study	Registration	Collecting and exchanging data is crucial for supporting road safety programs.
Chung A, et al. (2015)^47^	How accurate is accident data in road safety research? An application of vehicle black box data regarding pedestrian-to-taxi accidents in Korea	South Korea	(Mixed method study)	Registration	Collecting accurate data in police reports is a necessity of an accident information system. Using new technologies to record accident data online and using geographic information systems can help reduce errors in accident data collection.
Nie Q, et al. (2021)^44^	Electronic crash reporting: Implementation of the Model Minimum Uniform crash Criteria (MMUCC) and crash record life cycle comparison	USA	(Mixed method study)	Registration	The crash document form need to follow a coherent and complete workflow with MMUCC to make sure information layout, data exchange, data accuracy and quality.
Imprialou M, et al. (2019)^30^	Crash data quality for road safety research: Current state and future directions	UK	Review study	Registration	The accuracy and quality of data collected for exchange and the creation of accurate statistical reports play a crucial role in reducing accidents.
Rabbani M, et al. (2022)^31^	Road Accident Data Collection Systems in Developing and Developed Countries: A Review	Malaysia	Review study	Registration	Accident data registration plays a crucial role in the establishment of a crash information system, as it is essential for accident assessment, prediction, and analysis of consequences.
Sadeghi-Bazargani H, et al. (2020)^12^	Developing a national integrated road traffic injury registry system	Iran	(Mixed method study)	Registration	To develop an integrated national registry for Iran, measuring the content and core components of the conceptual model will assist researchers in enhancing the data collection tool for compiling the registry of road injuries.
Khorshidi A, et al. (2015)^45^	Traffic accidents information collection system in Iran, challenges and solutions	Iran	(Mixed method study)	Registration	This study highlighted the challenges and shortcomings of Iran's national road traffic information system. The use of software designed by PATAK enables the comprehensive utilization of data within the road traffic data collection system
Sadeghi-Bazargani H, et al. (2020)^39^	Road safety data collection systems in Iran: A comparison based on relevant organizations	Iran	(Qualitative study)	Registration	Integrating and developing a comprehensive evaluation system to collect a minimum data set is a critical requirement for any organization.
Mohammadi A, et al. (2016)^17^	Developing a Minimum Data Set for an Information Management System to Study Traffic Accidents in Iran	Iran	(Mixed method study)	Registration	Creating a Minimal Data Set (MDS) for a Road Traffic Crash Information System in Iran.
Stigson H, et al. (2021)^35^	Electric scooters accidents: Analyses of two Swedish accident data sets	Sweden	(Quantitative study)	Registration	This study illustrates the relationship between different registry types and emphasizes the importance of understanding how data elements influence outcomes
Hosseinzadeh A, et al. (2022)^48^	Data linkage for crash outcome assessment: Linking police-reported crashes, emergency response data, and trauma registry records	USA	(Mixed method study)	Data Exchange	By linking data from other sources, the available information can be greatly enhanced for road safety, data quality issues, and other issues.
Eskandari M, et al. (2022)^51^	Understanding the potential of emerging digital technologies for improving road safety	UK	Systematic review	Data Exchange	New technologies, including artificial intelligence and image processing, have been widely used to enhance road safety due to their ability to automatically collect, process, and exchange data.
Kamaluddin NA, et al. (2019)^42^	Matching of police and hospital road crash casualty records–a data-linkage study in Malaysia	Malaysia	(Quantitative study)	Data Exchange	The integration between the hospital and police information systems enhances the reliability and accuracy of the road traffic crash information system.
Soltani A, et al. (2024)^11^	Police and hospital data linkage for traffic injury surveillance	Iran	Systematic review	Data Exchange	Data exchange can provide valuable insights for investigating complex events, decision making and public health research.
Bakhtiari M, et al. (2013)^43^	Study of road traffic injuries risk factors by geographic information system (GIS)	Iran	(Quantitative study)	Data Exchange	The data integration of the pre-hospital system with Geographic Information Systems (GIS) helps identify accident-prone areas and improves the speed of providing healthcare services to the injured.

**Organizations involved in the National Road Accident Information Systems in Iran and selected countries**

Seven articles (37%) highlighted the involvement of multiple organizations, such as the Ministry of Health, Justice, Police, Insurance Companies, and Automobile Manufacturers, in promoting road safety.^2,5,6,13,22,25-27^ Given the involvement of numerous organizations in accident reduction, there is a need for a leading organization to coordinate their efforts. The leadership role of such organizations varies by country.^55^ For instance, in the United States, the Department of Transportation is responsible for managing road safety, while the National Highway Traffic Safety Administration (NHTSA) is tasked with collecting crash data from various sources.^56^ In England, the Department for Transport (DfT) oversees the national road safety strategy, managing the national road traffic information system through its statistical units and committees.^57^

In Malaysia, two articles (9%) reported that the Road Safety Department (RSD), established within the Ministry of Transport, serves as the leading institution in this regard.^9,57^ In Iran, two articles (9%) found that the Road Maintenance and Transportation Organization, as the guardian of the country’s transport system, works to enhance safety and ensure smooth traffic flow through the development of intelligent transportation systems.^58,59^ The Korea Road Traffic Authority (KoROAD), a government agency, supports the police by overseeing traffic management, driver licensing, and training.^48,60^ Additionally, the Swedish National Road Administration (SNRA) is the national authority responsible for the overall road transport system.^24^

**Data registration in the National Road Accident Information Systems in Iran and selected countries**

When registering crash data, a wide range of spatial and descriptive data must be considered.^61,62^ Two studies found that, in the United States, crash data must be collected by each state from various sources, including police accident reports, medical services, and state records such as vehicle and driver’s license registries.^31,44^ The collection of traffic crash data follows the Model Minimum Uniform Crash Criteria (MMUCC) dataset, which is designed to ensure data integrity.^44^ In the United Kingdom, the primary source of road crash data is the Set of Statistical (STATS19) database.^53,63^ The data elements in this database are based on a standardized dataset that is also used across European countries.^64^ Three articles (13.6%) reported that STATS19 includes data on all minor and severe traffic accidents reported by the police.^29-31^ Additionally, in England, the Hospital Episode Statistics (HES) database provides information on accident victims treated in hospitals.^29,30,65^

Since 1991, the Royal Malaysian Police (RMP) has been collecting crash data using the POL27 form. In 1997, the RMP introduced the Computerized Accident Recording System (CARS) to manage this data. CARS was designed to maintain records, analyze data, and generate accident statistics reports^66^ Two articles (9%) noted that, in Malaysia, each region has its own unique variables and reporting methods. To address these challenges, the CARSH data system was launched.^31,67^ In the Traffic Accident Analysis System (TAAS), crash data is initially recorded by police officers. The recorded information includes the severity of the crash, as well as details such as driver information, road specifications, and the accident location.^50,60,68^ In Sweden, crash data is collected through a web-based form^34,35^ The form consists of two pages; only the second page, which contains accident-related information, is entered into the STRADA system. The first page collects other information used by the police.^35,36^ In Iran, the traffic police collect and enter data using the Police 114KAM form on the web.^69^ One of the challenges in registering and collecting data on road traffic accidents is the gap between data elements and their insufficiency. Specifically, there is a need to define a minimum set of data elements to ensure accurate registration and data collection by the responsible organizations. ^17^

**Data exchange in the National Road Traffic Accident Information Systems in Iran and selected countries**

Data exchange facilitates the acquisition of complete and accurate accident reports.^70,71^ In the United States, the Crash Outcome Data Evaluation System (CODES) links crash data to medical record information, including Emergency Medical Services (EMS) data, which encompasses emergency department records, hospitalization details, and death certificates, through probabilistic record linkage.^70,72^ In the United Kingdom, linking the Hospital Episode Statistics (HES) and STATS19 databases enhances police data, providing a more comprehensive understanding of the accident environment and medical outcomes.^20,63^ Three studies (13.6%) found that the police, in collaboration with the Department for Transport (DfT), developed the Collision Recording and Sharing (CRASH) system to collect, validate, transfer, and store road accident reports according to police requirements.^9,31,66^ In Malaysia, the Centralized System for Road Accident, Safety, and Hazard Studies (CRASH) is connected via a local area network (LAN) to three central servers containing data from the Malaysian Institute of Road Safety Research (MIROS). The system is also linked to the Royal Malaysian Police (PDRM), the Road Transport Department (JPJ), and hospitals through the Internet.^31,67^

In South Korea, the Traffic Accident Analysis System (TAAS) utilizes an input management system to collect crash data, a statistical analysis system with Relational Online Analytical Processing (R-OLAP) tools, and a spatial analysis system equipped with Geographic Information Systems (GIS) to analyze spatial features. Additionally, the system includes a web service that provides real-time traffic incident information over the Internet. ^73^ The Safety and Traffic Reporting and Data Analysis (STRADA) system comprises two main databases: one for police data and another for hospital data.^34^ The STRADA database contains eight primary tables, including three specific police data tables, two hospital data tables, and three additional tables that include shared data from the police and hospital records.^23^ A table titled the "Matching Algorithm Results Report" is stored within the crash table, linking each report ID from the police and hospital tables to the corresponding crash ID.^34^ Two studies (9%) highlighted that in Iran, the lack of necessary connectivity between participating organizations, due to the differing nature of their databases, is one of the most significant challenges in obtaining accurate statistics.^28,69^ While Iran's traffic police system is based on Geographic Information System (GIS) technology, accident site information is recorded by experts in kilometers, and Global Positioning System (GPS) equipment is not utilized. The absence of precise geographical coordinates and communication limits the reliability of the data entered by traffic police experts.^43^

## Discussion

The current study aims to investigate the processes involved in the collection and exchange of crash data and to identify the lead organization overseeing the National Road Accident Information System. As established in the existing literature, efficient data collection and accessibility are critical to the effectiveness of a National Road Accident Information System.^74^ According to the study by Ehsani et al. (2023), in the United States, the design and implementation of a road traffic crash information system is not merely an intervention but a comprehensive set of strategies involving the participation of various organizations. The primary objective of such a system is to reduce traffic fatalities and serious injuries to zero, with an emphasis on equity. In this context, the study by Bliss et al. (2009) indicated that in the UK, a leading organization is responsible for identifying, creating, and funding government agencies, while also ensuring close collaboration between government and police agencies for the consistent reporting of crash data. In other words, by clearly defining the responsibilities of each organization, the leading entity facilitates the creation of an accident database to collect relevant data.^6^ Additionally, the study by Eusofe et al. (2017) in Malaysia found that the government established the Road Safety Department (RSD) as an independent entity under the Ministry of Transport (MOT), which was later recognized as the key organization responsible for road and traffic safety.^9^ The presence of a leading organization at the top level helps to coordinate various agencies and define their tasks.^75^ A range of crash data elements can provide valuable insights.^76^ In this regard, the Korea Transport Database (KTDB) was established in South Korea in 1999 to utilize essential crash data elements for the effective implementation of policies and projects related to road traffic accidents.^77^ The KTDB contains a comprehensive range of accident data types.^78^

In the study conducted by Amini et al. (2013) in Iran, the diverse range of road users and their varying information needs has made the classification and presentation of data a significant challenge^79^ According to the study by Mohammadi et al. (2016), in Iran, the foundation for collecting appropriate data on the causes of road traffic accidents and providing information to relevant organizations is the establishment of a minimum set of data that enables comparison between organizations.^17^ An investigation of the Iranian police database revealed that the absence of a data element for a 30-day post-mortem follow-up is evident in all reports, leading to inconsistencies when comparing data sources with those from other countries for international reports^31^ Additionally, the database of the Center for Medical Accidents and Emergencies lacks information on the condition of traffic-injured individuals after their transfer to the emergency department.^69^

Organizational structures and data registration are crucial, but the interconnection between databases is essential for generating comprehensive reports and enhancing managerial awareness across all countries. In this context, Abdulhafedh A. et al. (2017) conducted a study in the United States, which indicated that the CODES system, developed by the National Highway Traffic Safety Administration (NHTSA), is designed to link crash data with injury data collected from emergency services.^19^ Recognizing the significance of data linkage, the Guidance for Developing Highway Safety Plans (GHSA) and NHTSA introduced a web-based tool, "Mapping of Police Reports to MMUCC," to help U.S. states assess the compliance of Police Accident Reports (PAR) data with the Model Minimum Uniform Crash Criteria (MMUCC). Additionally, Nie Q et al. (2021) highlighted that integrating driver record systems has significantly contributed to the generation of high-quality reports with minimal errors.^44^ According to the Road Safety Strategy Framework Report (2021) in England, police reports did not consistently register the full range of injuries in terms of severity. Only 40 percent of police reports aligned with the injuries of seriously injured individuals as recorded in hospital registers. However, through the integration of Hospital Episode Statistics (HES) data and the development of a new reporting system, a more comprehensive understanding of injury severity and its consequences emerged in police reports.^80^ Similarly, in Malaysia, accident details are primarily provided by the Royal Malaysian Police (RMP) reports, while information on road injuries and fatalities is recorded in hospital registers. Analysis of data from both sources revealed that out of 7,625 accidents documented by the police and 362 recorded by hospitals, only 311 accidents were common to both reports, indicating incomplete data sharing.^31^ In Sweden, the connection between the police database and hospitals involves the police transmitting information from the police database to STRADA via a web-based client after a report is made.^23,81^ In the hospital database, nurses input patient form data along with medical information—such as doctor's notes, ambulance reports, X-ray scans, etc.—into the web client, which is then sent to STRADA.^23^

A study by Sadeghi et al. (2020) in Iran highlighted the potential benefits of data integration, specifically the connection between Electronic Medical Records in hospitals and police data. This integration has led to improved coordination, enhanced road traffic injury statistics, more accurate predictions of road casualties, and the development of strategies to prevent future incidents.^12^ However, there is limited alignment and overlap between the reports from the accident and emergency management center and the traffic police.^69^ In Iran, to implement a National Road Traffic Information System in a centralized and cross-sectoral manner, further research is needed on data exchange and the role of the leading organization. The findings of the current study are crucial for enhancing road safety and increasing stakeholder awareness.

## Conclusion

Based on the findings of the current study, organizations such as the National Highway Traffic Safety Administration (NHTSA) in the United States, the Department for Transport (DfT) in the United Kingdom, the Swedish Transport Administration (SNRA) in Sweden, the Korea Road Traffic Authority (KoROAD) in South Korea, and the Road Safety Department (RSD) in Malaysia are identified as key leading organizations. Furthermore, the study confirms that the registration of essential data elements, based on the global standard minimum data set, and the sharing of data across different organizations facilitate access to critical information for managers and policymakers. This, in turn, can lead to the development of more effective strategies and informed decision-making. Additionally, policymakers and researchers can utilize the findings of this study to implement more targeted interventions aimed at reducing the wide range of road injuries and fatalities.

**Authors Contributions:**

The inclusion and exclusion criteria were defined by AD, MA, and FA. HE and AH conducted the screening of articles, while AD, MA, and FA managed the selection of articles. MA prepared the initial draft of the manuscript, which was subsequently revised and assessed for quality by HE, AH, and AD. Additionally, MA, HE, and AH contributed to the conceptualization of the study. AD, FA, HE, and AH provided editorial feedback on all drafts. All authors reviewed and approved the final version of the manuscript.
